# Exploring the protective role of metformin and dehydrozingerone in sodium fluoride-induced neurotoxicity: evidence from prenatal rat models

**DOI:** 10.1007/s13205-024-04175-4

**Published:** 2025-01-08

**Authors:** Tejas Ahuja, Farmiza Begum, Gautam Kumar, Smita Shenoy, Nitesh Kumar, Rekha R. Shenoy

**Affiliations:** 1 Department of Pharmacology, Manipal College of Pharmaceutical Sciences, Manipal Academy of Higher Education, Manipal, Karnataka 576104 India; 2 Department of Pharmacology, Vaagdevi Pharmacy College, Bollikunta, Warangal, Telangana 506005 India; 3 School of Pharmacy, Sharda University, Greater Noida-201306, Uttar Pradesh, India; 4 Department of Pharmacology, Kasturba Medical College, Manipal Academy of Higher Education, Manipal, Karnataka 576104 India; 5 Department of Pharmacology and Toxicology, National Institute of Pharmaceutical Education and Research, Vaishali, Hajipur, Bihar, 844102 India

**Keywords:** Sodium fluoride, Prenatal development, Neurodevelopmental toxicity, Metformin, Dehydrozingerone, Oxidative stress, Cognitive impairment

## Abstract

This study is aimed at evaluating the neurotoxic effects of chronic exposure of sodium fluoride (NaF) in developmental stages in rat using prenatal models. NaF (100 ppm, orally) dosing via drinking water was given to pregnant rats in disease group. In the treatment groups, Metformin & Dehydrozingerone (DHZ) (200 mg/kg) were administered orally along with NaF, and the dosing was continued throughout the gestation and lactation periods to the pups until the end of experiment. Behavioural studies like Novel Object Recognition Test (NORT), Open Field & Actophotometer test and biochemical estimations like Acetylcholinesterase (AchE), Glutathione (GSH), Malondialdehyde (MDA) were conducted on animals followed by histopathological image analysis. It was observed that NaF exposure significantly decreased learning, memory and locomotor ability (at p < 0.05, p ≤ 0.01) in rat pups and was also able to induce anxiety like behavior. Levels of AchE (p ≤ 0.001) and MDA (p ≤ 0.01, p ≤ 0.001) was found to be significantly elevated and GSH levels were significantly decreased (p ≤ 0.01, p ≤ 0.001) in hippocampus and frontal cortex in the disease group. Histopathological image analysis showed presence of degenerated neurons in hippocampus of disease group. From this study, it was observed that treatment with Metformin and DHZ, was able to significantly ameliorate the cognitive impairments, improve the condition of oxidative stress and decrease neuronal degeneration in NaF fed rat pups. These results established the protective role of Metformin and DHZ in NaF induced neurodevelopmental toxicity with particular emphasis on their antioxidant properties.

## Introduction

Fluoride is one of the most important inorganic anions that is widely used in clinical practice and has been added in many water sources to prevent problems like osteoporosis and dental caries. Fluorination of water was considered as one of the major achievements of the past century and it is considered by health workers to be necessary for normal human development. However, for the past few years it has been observed that consumption of fluoride in excess amounts has detrimental effects on normal functions in the body. It was already known to cause toxicity in teeth and bones (skeletal fluorosis) and many studies conducted recently have also reported its toxicity in central nervous system, mostly in children (Choi et al. 2012; Aoun et al. 2018). As a result, many epidemiological studies have been conducted all over the world and it was found that in areas where the fluoride levels in water were higher than the permitted amount (mostly > 1.2 mg/L), there was a sharp decline in intelligence and memory in children below the age of 4 (Choi et al. 2012; Aoun et al. 2018; Kanduti et al. 2016).

A study reported that fluoride was able to easily cross the placental barrier during pregnancy and can also permeate through the blood brain barrier in early life stages when it is not fully developed (Madhusudhan et al. 2010). Various studies have reported different mechanisms like reduction in levels of nicotinic and muscarinic receptors, autophagy and apoptosis in neurons, decreased glucose consumption, inhibition of enzymes involved in the generation of energy and transmission of synapse, mitochondrial dysfunction and increased oxidative stress leading to inflammation and neuronal cell death (Tu et al. 2018; Niu et al.^2018^, ^2009^; Yan et al. 2016; Basha et al. 2010). Increase in oxidative stress was reported to be one of the main mechanisms of fluoride induced neurotoxicity (Basha et al. 2010; Liu et al. 2014) Based on these inferences, various natural compounds having antioxidant properties, for example curcumin, aloe vera, quercetin, epigallocatechin gallate etc. have been studied for their protective role in NaF induced neurotoxicity in prenatal model (Garcia et al. 2017; Wang et al. 2012).

Metformin is a first line drug in type-2 diabetes and beneficially affects lipid profile, decreased inflammatory cell attachment (Adhesion) to vascular endothelium, exerts anti-inflammatory property, anti-apoptotic action and anti-oxidative property (Shanmugam et al. 2018; Dehkordi et al. 2019). Apart from its therapeutic effects in diabetes, it has been extensively studied for its neuroprotective effects in the pathogenesis of Alzheimer’s where it is shown to revert learning and memory impairments. One of the major mechanisms by which it can provide protection against neurodegenerative disorders is due to its antioxidant properties. A study evaluated the antioxidant property of metformin where it was observed that oxidative stress played a major role in pentylenetetrazole (PTZ) induced seizure and treatment with metformin was able to ameliorate the increase in stress by decreasing MDA levels and elevating GSH levels in the brain (Zhao et al. 2014; Ashabi et al. 2015). DHZ is a half analogue of curcumin and like curcumin it exhibits antioxidant, anti-inflammatory, cardio-protective, and chemo-protective effects (Rajakumar et al. 1994; Kim et al. 2013). The aim of this study was to evaluate the possible effects of NaF toxicity in the prenatal stage on behaviour of rat pups and to check whether Metformin and DHZ can prevent/treat this toxicity by reducing the oxidative stress with special emphasis on hippocampus and frontal cortex.

## Materials and methods

### Chemicals

Metformin and NaF were purchased from Kasturba Medical College’s Pharmacy section in Manipal, Karnataka, India. The following materials were obtained: μQPRO BCA Protein Estimation Kit from Cyanagen, Carboxy Methylcellulose (CMC), Acetone, Sodium hydroxide, Vanillin (for the synthesis of Dehydrozingerone), and antibodies against AMPK & P-AMPK from Elabscience life sciences.

### Animals

Sixteen Female Wistar Rats along with eight Male Wistar Rats were procured from the Central Animal Research Facility, MAHE, Manipal. All animals were housed at controlled room temp (23 ± 2 °C), controlled humidity (50 ± 5%) along with free access to food and water *ad libitum*. Light and dark cycles of 12:12 h was maintained. All the guidelines according to Committee for the Purpose of Control And Supervision of Experiments on Animals (CPCSEA) and Institutional Animal Ethics Committee (IAEC) were followed while performing this study. This study was approved by Institutional animal ethics committee, KMC Manipal with Reg. No. IAEC/KMC/130/2020.

### Experimental design

On day 1 of pregnancy, female rats were divided into four groups. Experimental Design and dosage were selected according to De Oleveira et al. 2016 & Anusha et al. 2016. Group I was normal control which received tap water, Group II was disease control which received NaF (100 mg/L) dissolved in distilled water (Niu et al. 2009; Zhao et al. 2019). Group III was treatment that received metformin (200 mg/kg) p.o. (De oleivera et al. 2016) and Group IV was treatment which received dehydrozingerone (200 mg/kg) p.o (Anusha et al. 2016) along with NaF in drinking water. NaF, Metformin & DHZ were suspended in 0.5% Carboxy Methyl Cellulose and was given orally to pregnant rats as gestational exposure. This was continued until the pups were born, till the end of study. NaF was supplied to the pups via mother during the lactation period. After 21st day NaF was directly administered orally to the rat pups. Dose of Metformin and DHZ was selected based on the previous studies where at 200 mg/kg they showed significant anti-oxidant activity (De oleivera et al. 2016; Anusha et al. 2016). Total of three litters were randomized for the study where each litter had an average of eight pups. Pups aged 7–11 days were checked for their neonatal reflexes. After the behavioral studies, animals were sacrificed, and hippocampus and frontal cortex were collected and stored for further analysis.

### Timeline of study

**Fig. 1:**
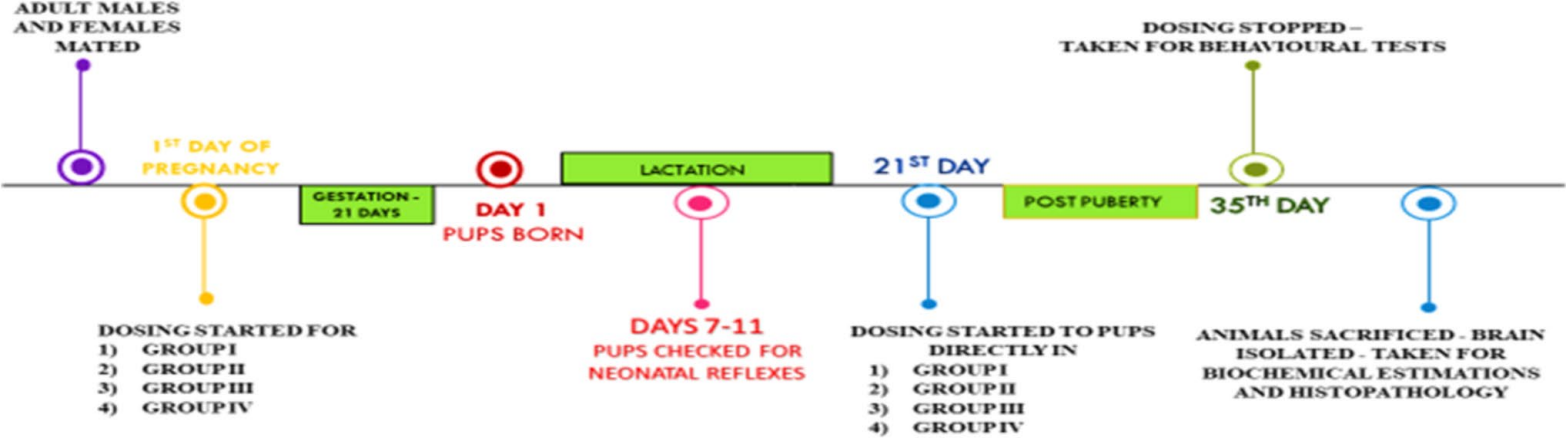
Timeline of study. Group I was Normal control which received tap water, Group II was disease control animals which received NaF (100 mg/L) dissolved in distilled water. Group III was treatment group that received metformin (200 mg/kg) p.o. and Group IV was treatment group which received dehydrozingerone (200 mg/kg) p.o. along with NaF via drinking water

Figure 1 depicts the overall study, groups involved and methods to be performed.

## Methodology

### Neonatal reflexes

Series of tests were conducted to check the development of normal reflexes in rat pups of disease and treatment groups during gestational period when compared to normal group pups. Various in-house techniques were used to perform these tests and the tests were conducted over a duration of five days (from day 7 to 11).

### Righting reflex

The righting reflex test started on post-natal day 3 (PD3). All four paws were erect when we firmly held the pup in the supine position. Timer was set immediately after releasing the pup. A pup is said to have successfully completed a full roll or flip onto all four paws when each paw is parallel to the body. To accomplish this, each pup was given a maximum of 15 seconds (s). The time taken by the pup to return to its fore limbs when it was placed with its back facing towards the flat surface of table was recorded (Cut-off time 30 s) (Nguyen et al. 2012).

### Forelimb grasp

Forelimb grasp reflex test was performed on PD3. A 16-inch needle barrel was placed in forepaw of the pup and lightly pressed with hands. To make sure that the pups made contact and felt the needle barrel, we applied gentle pressure that displaced the forepaw slightly. When we grasped, the entire digit flexed around the barrel. When both forepaws successfully acquired this reflex, they would hold onto the barrel for two consecutive days. Pups would grasp the needle barrel (16-gauge) when it was rubbed on their forepaw (Baharnoori et al. 2012; Nguyen et al. 2012).

### Grip strength test

The hind-limb grab reflex test was performed on PD3. A blunt rod was placed on each hind-paw and lightly pressed with hands. To make sure the pups made contact and felt the rod, gentle pressure was applied that displaced the hind-paw slightly. Flexion of the digits around the rod signified a successful grip. When both hind-paws held onto the rod for two consecutive days, the reflex was successfully learnt (Time– 5 s) (Nguyen et al. 2012).

### Negative geotactic test

On an inclined surface of 45°, the pups were placed with their head facing downside, and the time taken for the pup to turn around and move towards the upper end of inclined surface was noted. (Time limit 180 s) (Baharnoori et al.2012 2102; Nguyen et al. 2012).

### Novel object recognition test

The task consists of three different phases i.e., Acclimatization or habituation phase, familiarization phase and Test phase (also called choice trial) with an inter-trial interval of 12 h, 3 min duration each. Bandicam software was used to analyze these recordings for further analysis (Mathiasen et al. 2010). Recognition Index (Ri) refers to, time spent by the animal discovering or exploring the novel object divided by the total exploration time i.e., RI = *B/* (*A1* + *B*). Discrimination Index (Di) refers to the difference between exploration period of novel object (B) and the familiar object (A1) and the total exploration time during test phase i.e., DI = *B* – *A1*/ (*A1* + *B*) (Pathak et al. 2020).

### Locomotor test

The locomotor ability of the animals was assessed using actophotometer. Rat was placed in the center of platform and was allowed to move freely in the apparatus for 5 min. Locomotor activity was recorded using digital counter on the apparatus. After 30 min of dosing with treatment, rat was placed in the apparatus for recording the locomotor activity (Pathak et al. 2020).

### Open field locomotor test

Behavioral test was used to test various parameters like locomotor, anxiety, exploration and in some cases hyperactivity ( Sestakova et al. 2013; Chioca et al. 2008). The floor was divided into 9 equal squares. The rat was kept in the central square & was allowed to explore the open field for 6 min.

### Biochemical estimations

Oxidative stress markers like Lipid Peroxidation (LPO) by MDA estimation (Konings et al. 1979), Reduced Glutathione** (**GSH) (Rahman et al. 2007), AchE levels (Ellman et al. 1961) and total protein were estimated in the frontal cortex and hippocampus regions of brain using UV Spectrophotometric and colorimetric methods.

### Histopathological image analysis

The brain samples were immediately stored in neutral formalin buffer for Hematoxylin and Eosin (H&E) staining. Brain tissues stored in neutral buffered formalin were dehydrated using a graded ethanol series. 5-micron sections of paraffin embedded tissue blocks were made on microtome. Later, staining of the tissue sections was done using Hematoxylin & eosin stain. Analysis was done using Olympus microscope.

### Statistical analysis

Graph Pad prism 8.4.2 Version was used for statistical analysis. Data was expressed as Mean ± SEM for three samples each and was analyzed using One-way ANOVA followed by Bonferroni’s Post hoc test.

## Results

### Neonatal reflexes

#### Righting reflex

**Fig. 2:**
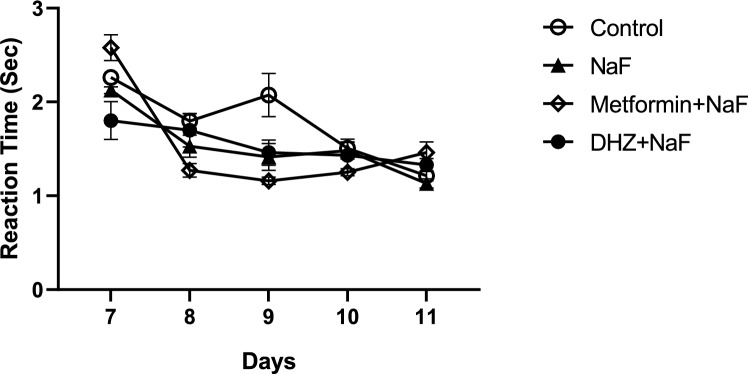
Effect of NaF and NaF + Treatment (Metformin and DHZ) on Righting reflex in pups of days 7–11 for NaF induced neurodevelopmental toxicity. Data was represented as Mean ± SEM. Analysis of variance (one way) & Bonferroni’s post hoc test was used for analysis (P < 0.05)

Exposure to NaF during pregnancy did not affect the righting reflexes in the pups of disease group as the reaction time was not significantly different from that of pups in normal control group (p < 0.05). Also, treatment with both Metformin & DHZ did not significantly improve the reflex compared to disease group from post-natal day 7–11 (p < 0.05) (Fig. 2).

#### Forelimb grasp test

**Fig. 3:**
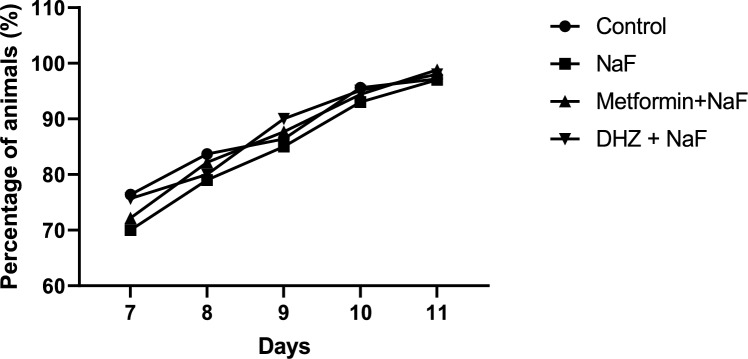
Effect of NaF and NaF + Treatment (Metformin and DHZ) on forelimb grasp reflex in pups of days 7–11 for NaF induced neurodevelopmental toxicity. Data was represented as Mean ± SEM. Analysis of variance (one way) & Bonferroni’s post hoc test was used for analysis (P < 0.05)

No Significant Difference in the percentage of animals showing forelimb grasp reflex was observed among treatment groups when compared with normal control and NaF from post-natal day 7–11 (p < 0.05) (Fig. 3).

#### Grip strength test

**Fig. 4:**
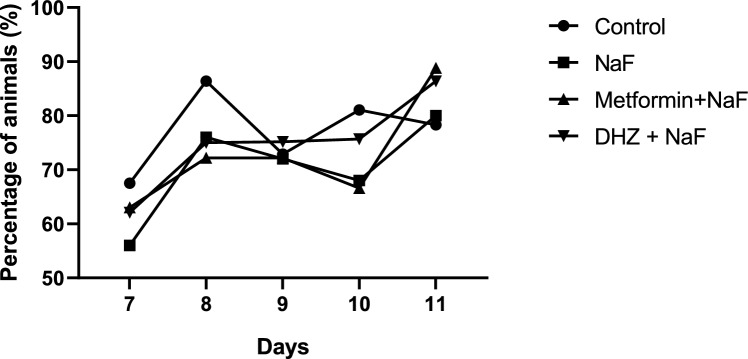
Effect of NaF and NaF + Treatments (Metformin and DHZ) on Grip strength reflex in pups of days 7–11 for NaF induced neurodevelopmental toxicity. Data was represented in the form of Mean ± SEM. Analysis of variance (one way) & Bonferroni’s post hoc test was used for analysis (p < 0.05)

No Significant Difference in the percentage of animals successfully showing grip strength reflex was observed among treatment groups when compared with normal control and NaF from post-natal day 7–11 (p < 0.05) (Fig. 4).

#### Negative geotactic reflex

**Fig. 5:**
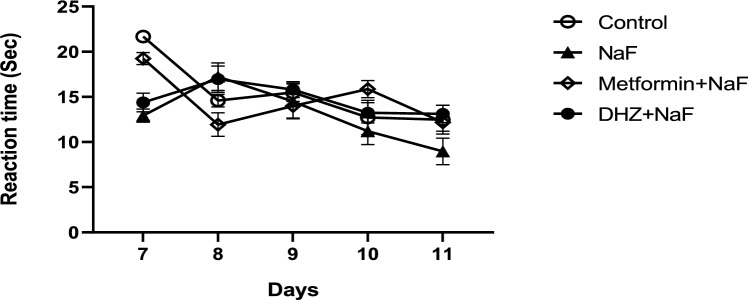
Effect of NaF and NaF + Treatments (Metformin and DHZ) on Negative geotactic reflex in pups of days 7–11 for NaF induced neurodevelopmental toxicity. Data was represented in the form of Mean ± SEM. Analysis of variance (one way) & Bonferroni’s post hoc test was used for analysis (p < 0.05)

Prenatal exposure to NaF did not affect the negative geotactic reflex in the pups of disease group as the reaction time was not significantly different from that of pups in normal control group (p < 0.05). Also, treatment with both Metformin and DHZ did not significantly improve the reflex compared to disease group from post-natal day 7–11 (p < 0.05) (Fig. 5).

### Novel object recognition test

#### Recognition index, discrimination index & exploration time of novel object

**Fig. 6:**
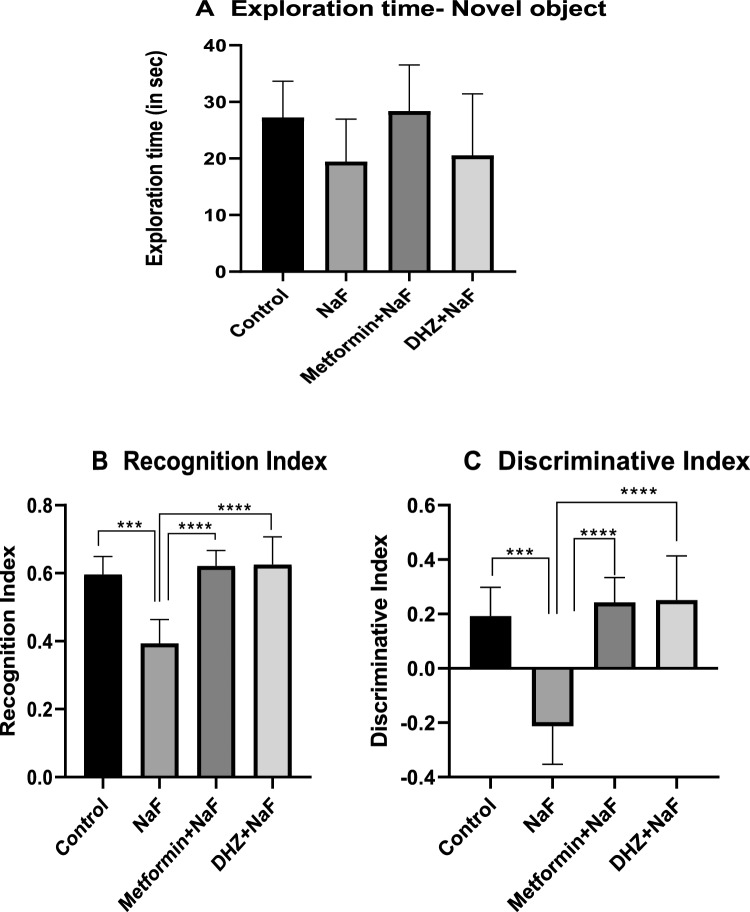
Effect of NaF and NaF + Treatments (Metformin & DHZ) on Recognition index **B**, Discrimination index **C** & Exploration time of novel object **A** in prenatal model for NaF induced neurodevelopmental toxicity. Data was represented in the form of Mean ± SEM. Analysis of variance (one way) & Bonferroni’s post hoc test was used for analysis (P < 0.05). **B**: ***—Significant difference was observed among normal control group and disease (NaF only) group at p ≤ 0.001, ****—Significant difference was observed among disease (NaF only) and treatment groups at p ≤ 0.0001. **C**: **** -Significant difference observed between disease (NaF only)-normal control and disease (NaF only)-treatment groups at p ≤ 0.0001

No significant difference in the exploration time of novel object was observed between normal control and NaF group as well as between NaF group and both the treatment groups at P < 0.05. Significant difference (p ≤ 0.001) was seen in recognition index of novel object between normal control and disease group. Treatment with Metformin and Dehydrozingerone in prenatal model significantly increased (p ≤ 0.0001) recognition indices of novel object. Significant decrease was observed in discrimination between familiar and novel objects between normal control and NaF groups at p ≤ 0.001. Treatment with Metformin and Dehydrozingerone in NaF treated rats significantly increased discrimination index at p ≤ 0.0001 (Fig. 6A–C).

#### Locomotor test

**Fig. 7:**
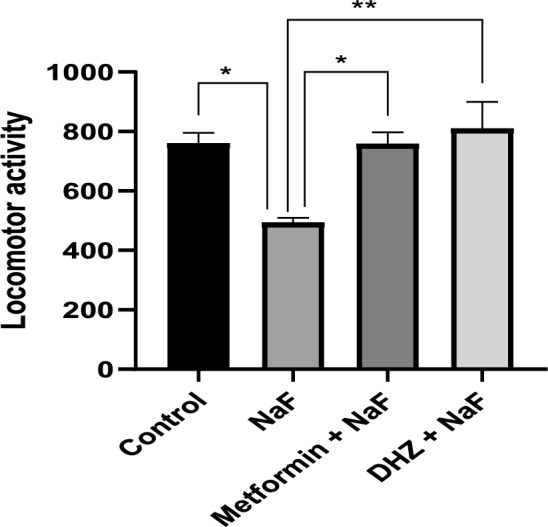
Effect of NaF and subsequent treatment with Metformin and DHZ on locomotor activity of pups in prenatal model for NaF induced neurodevelopmental toxicity. Data was represented in the form of Mean ± SEM. Analysis of variance (one way) & Bonferroni’s post hoc test was used for analysis(P < 0.05) * -Significant difference was observed among normal control-disease (NaF only) groups and disease (NaF only) -Metformin group at p ≤ 0.05, **—Significant decrease was observed among disease group (NaF only) -DHZ treated group at p ≤ 0.01

Prenatal exposure to NaF in rat pups significantly decreased the locomotor activity of pups compared to the rats in normal control group (at p ≤ 0.05). Treatment of NaF exposed rats with Metformin and DHZ significantly increased locomotor activity in pups (at p ≤ 0.05) (Fig. 7).

### Open field locomotor test

#### Number of line crossings

**Fig. 8:**
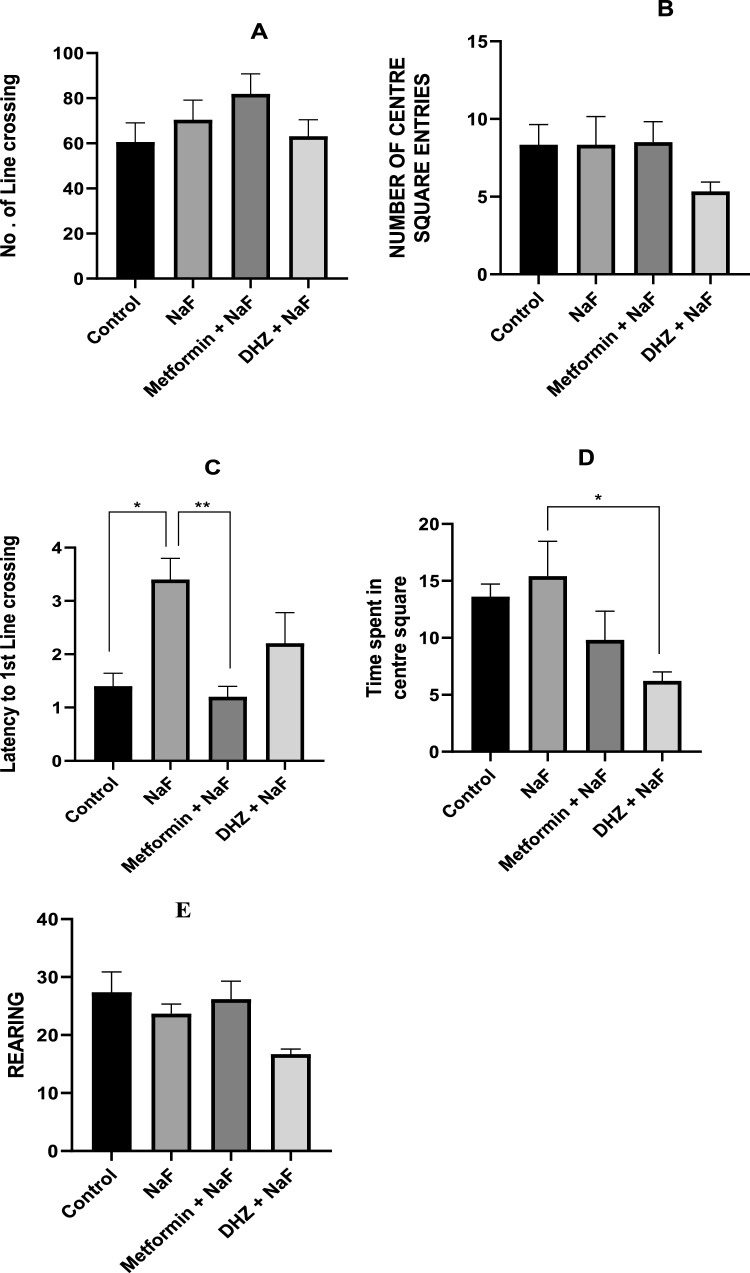
Effect of NaF and subsequent treatment with Metformin and DHZ on changes in locomotor activity in open field test for NaF induced neurodevelopmental toxicity. Data was represented in the form of Mean ± SEM. Analysis of variance (one way) & Bonferroni’s post hoc test was used for analysis(P < 0.05). **C**: *- Significant difference was observed among normal control group and disease (NaF only) group at p ≤ 0.01, ** Significant difference was observed among disease (NaF only) and NaF + Metformin group at p ≤ 0.006, **D**: *- Significant difference was observed among disease (NaF only) and NaF + DHZ group at p ≤ 0.04

No significant change (P < 0.05) in number of line crossing was observed in disease group as compared to the normal control group. Treatment groups also showed no significant change in line crossing compared to both disease and normal control group (Fig. 8A).

#### Number of center square entries

Exposure of animals to NaF showed no significant change in the number of center square entries in comparison to normal control group and metformin treatment. Even though DHZ group decreased the number of entries, the difference with respect to disease group was not significant at p < 0.05 (Fig. 8B).

#### Latency to first line crossing

NaF exposure in disease group significantly increased the latency of animals to first line crossing compared to normal control animals indicating decreased locomotor activity and presence of anxiety like behavior. Metformin treatment was able to significantly reverse this effect (significant at p ≤ 0.01), but a significant decrease was not observed in DHZ treated rats (at p < 0.05) (Fig. 8C).

#### Time spent in center square

A slight increase in time spent in center square was seen in disease group compared to the normal control, however the change was not significant at p < 0.05. Both Metformin and DHZ treatments reduced the time considerably, but significant difference compared to NaF treated group was reported only for DHZ (at p < 0.05) (Fig. 8D).

#### Rearing

No significant change in rearing was observed in both disease and treatment groups compared to normal control. DHZ treatment was able to decrease the number of rearing in group IV, however the change was not significant at p < 0.05 (Fig. 8E).

### Biochemical estimations

#### Acetylcholinesterase activity in hippocampus

**Fig. 9:**
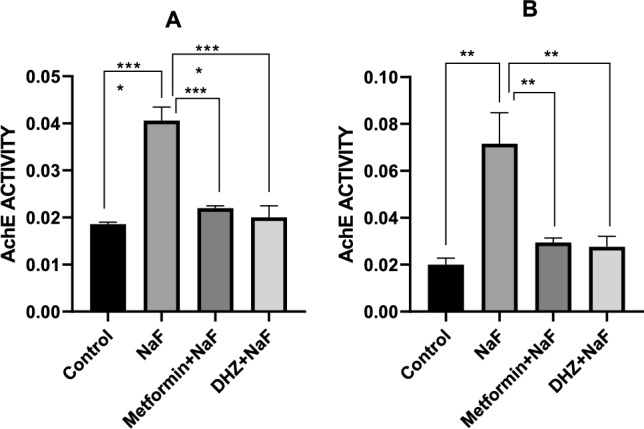
Effect of NaF and NaF + Treatments (Metformin and DHZ) on acetylcholinesterase activity in Hippocampus **A** & Frontal cortex **B** of pups in prenatal model for NaF induced neurodevelopmental toxicity. Data was represented in the form of Mean ± SEM. Analysis of variance (one way) & Bonferroni’s post hoc test was used for analysis(P < 0.05). **A**: ***—Significant Difference observed between normal control and disease (NaF only) groups and disease (NaF only) and treatment groups at p ≤ 0.001

Prenatal exposure to NaF in pups significantly increased acetylcholinesterase activity in hippocampus when compared to normal control group (at p ≤ 0.001). Metformin and DHZ significantly decreased acetylcholinesterase activity in rat pups when compared to disease group (at p ≤ 0.001) (Fig. 9A).

Acetylcholinesterase activity in Frontal Cortex

Prenatal exposure to NaF in rat pups led to an increase in Acetylcholinesterase activity in the frontal cortex, however the difference compared to the normal control group was not significant at p < 0.05. Treatment of rat pups with metformin and DHZ restored the Acetylcholinesterase levels to normal, however the decrease as compared to NaF group was not significant at p < 0.05 (Fig. 9B).

### Lipid peroxidation assay

#### Hippocampus

**Fig. 10:**
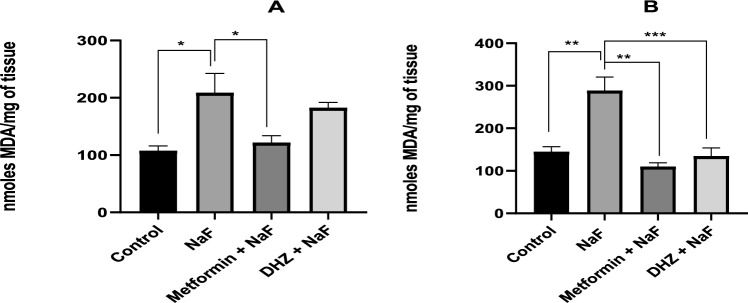
Effect of NaF and NaF + Treatments (Metformin and DHZ) on Malondialdehyde (MDA) levels in Hippocampus (Fig. 10A) & Frontal Cortex (Fig. 10B) of pups in prenatal model for NaF induced neurodevelopmental toxicity. Data was represented in the form of Mean ± SEM. Analysis of variance (one way) & Bonferroni’s post hoc test was used for analysis(P < 0.05). **A**: *—Significant Difference observed among Normal control-Disease (NaF only) group and disease (NaF only)-Metformin treated group at p ≤ 0.05, **—Significant Difference observed among Normal control-Disease (NaF only) group and disease (NaF only)-Metformin treated group at p ≤ 0.01 **B**: ***—Significant decrease was observed between disease (NaF only) group and DHZ treated group at p ≤ 0.001

NaF exposure in the prenatal models, led to a significant increase in MDA levels in hippocampus (p ≤ 0.05) when compared to the normal control group. This increase was significantly reversed by treating the animals with Metformin. Although, DHZ treatment reduced the MDA level, the decrease was not significant at p < 0.05 (Fig. 10A).

#### Frontal cortex

MDA levels in the frontal cortex of disease group was significantly elevated when compared to the normal control group (at p < 0.01). This elevation was reversed in groups III and IV, where DHZ treated rats showed a more significant reduction compared to the Metformin treated rats (Significant at p < 0.001 and p < 0.01 respectively) (Fig. 10B).

### Reduced glutathione assay

#### Hippocampus

**Fig. 11:**
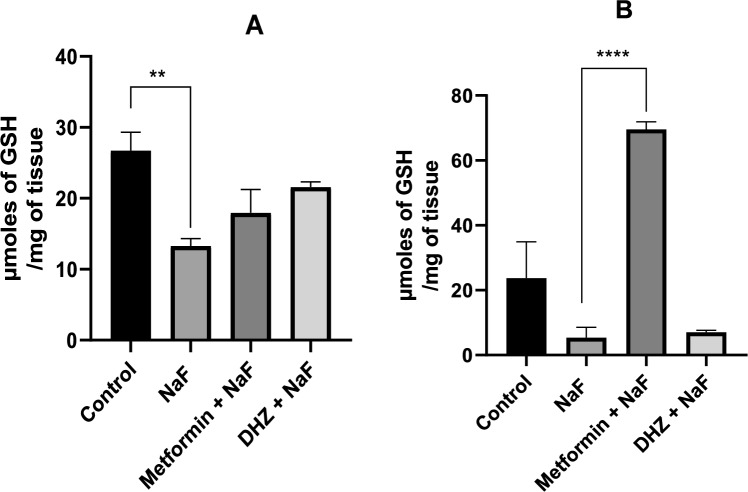
Effect of NaF and NaF + Treatments (Metformin and DHZ) on GSH levels in Hippocampus **A** & Frontal cortex **B** of pups in prenatal model for NaF induced neurodevelopmental toxicity. Data was represented in the form of Mean ± SEM. Analysis of variance (one way) & Bonferroni’s post hoc test was used for analysis(p < 0.05). **A**: **—Significant Difference observed among normal control and disease (NaF only) groups at p ≤ 0.01, **B**: ***—Significant Difference observed among disease (NaF only) groups and Metformin treated group at p ≤ 0.001

In NaF treated rats, glutathione levels were significantly decreased when compared to normal control group (at p ≤ 0.01). Even though, treatment with Metformin & DHZ increased the GSH levels in hippocampus, the difference between treatment and disease groups was not found to be significant at p ≤ 0.05 (Fig. 11A).

#### Frontal cortex

In frontal cortex of pups in prenatal models, exposure to NaF decreased the GSH levels when compared to the normal control group, however this decrease was not significant at p ≤ 0.05. DHZ treatment in this model was not able to increase GSH levels significantly. However, a highly statistically significant increase in levels were observed in rats treated with Metformin (significant at p ≤ 0.001) compared to NaF treated group (Fig. 11B).

#### Effect on body weight

**Fig. 12:**
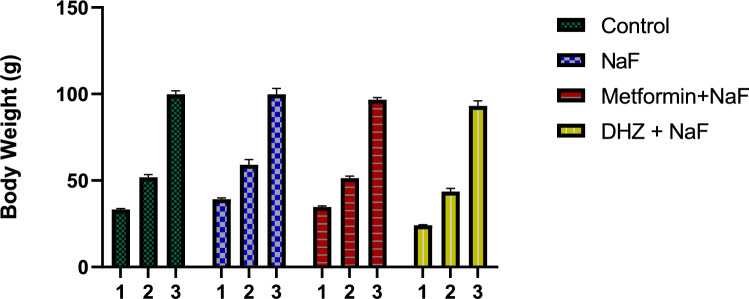
Effects of NaF and NaF + Treatments (Metformin and Dehydrozingerone) on body weights in prenatal model for NaF induced neurodevelopmental toxicity. Data was represented in the form of Mean ± SEM. Analysis of variance (one way) & Bonferroni’s post hoc test was used for analysis (P < 0.05). *Significant Difference was observed among disease (NaF only) and Metformin groups at p ≤ 0.05 ****Significant Difference was observed among disease (NaF only) and DHZ treated groups at p ≤ 0.0001

Body weight of pups were taken after completion of lactation period (21 days) and there was no significant difference observed between the weights of rats in disease and normal control group (p < 0.05). On the other hand, pups in treatment groups had significantly lower body weights compared to the disease group where DHZ group had the lowest mean weights among all groups (Fig. 12).

#### Histopathological image analysis

**Fig. 13:**
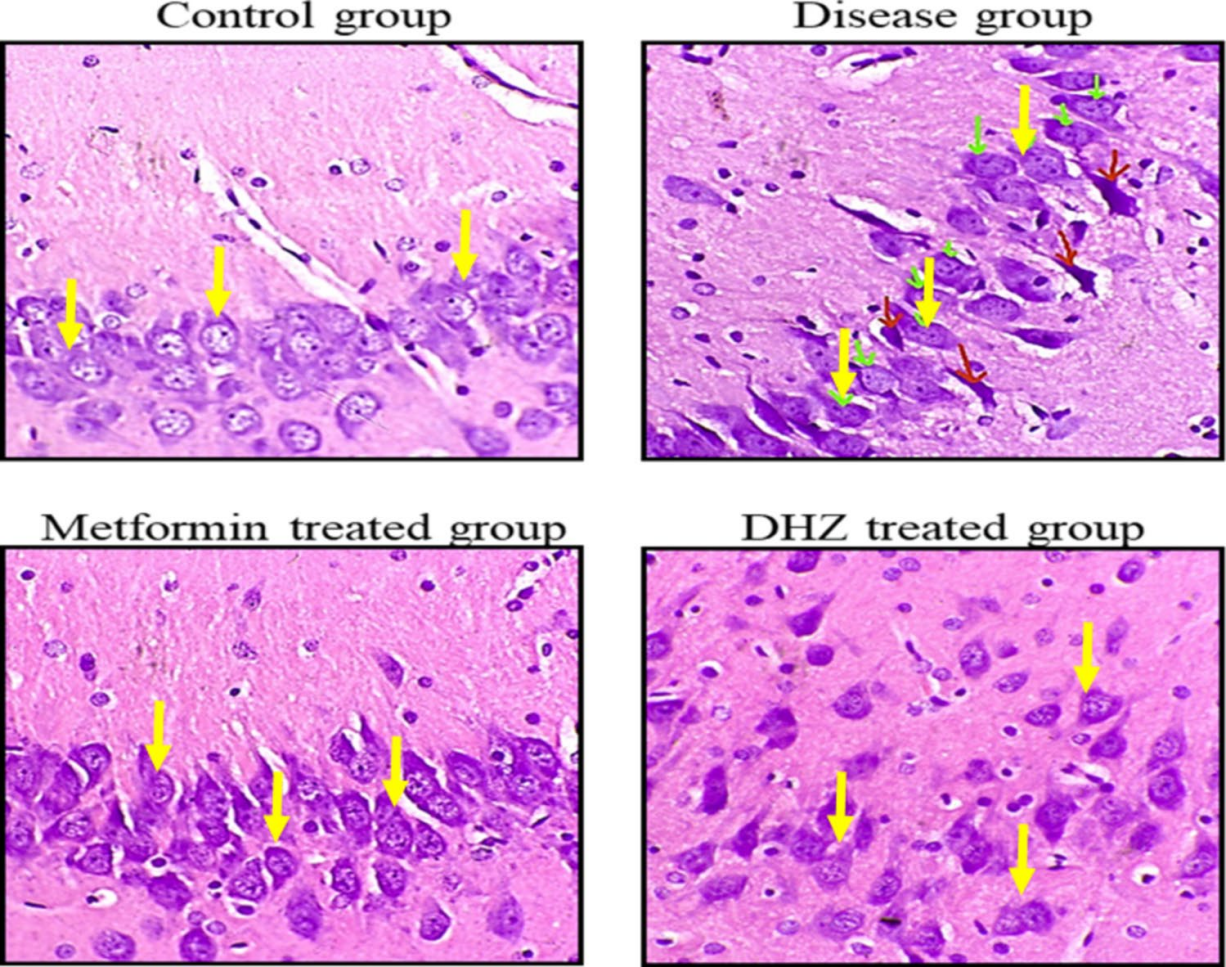
Photomicrographs of CA3 region of hippocampus in different groups (H&E stain). Arrows indicate degenerated neurons in disease group (NaF only) and healthy neurons with pale and round nucleus, well defined nuclear boundary and prominent nucleoli in normal control and treatment groups seen in hippocampus

**Fig. 14:**
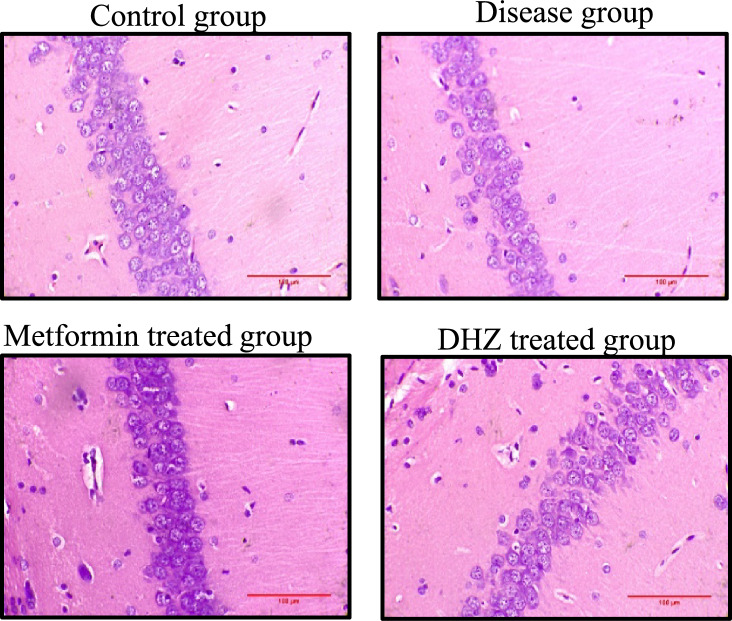
Photomicrographs of CA1 region of hippocampus showing healthy neurons in different groups (H&E stain). No significant histological changes observed

**Fig. 15:**
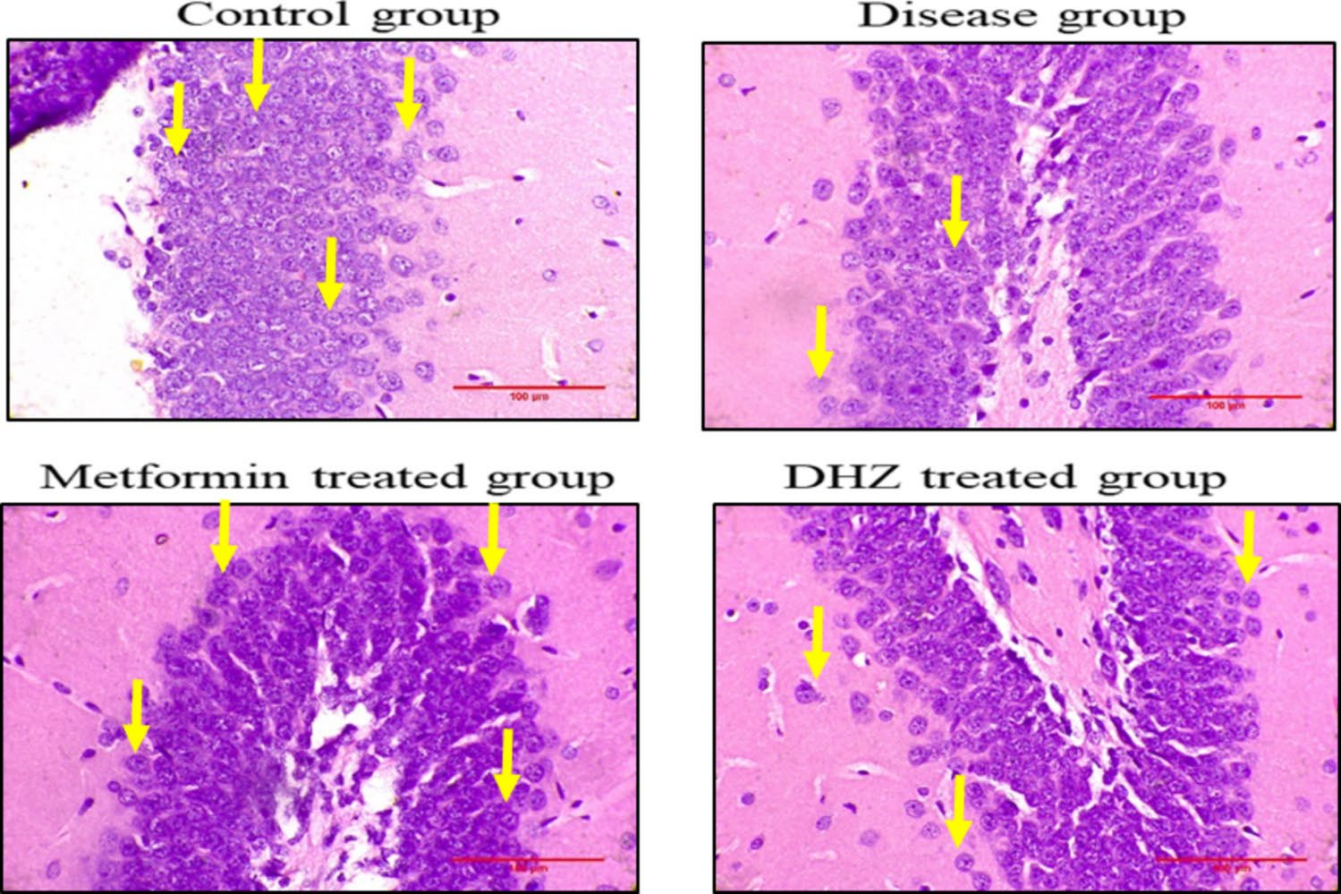
Photomicrographs of Dentate gyrus in different groups (H&E stain). Arrows indicating few degenerated neurons which are darkly (basophilic) stained, with shrunken and fragmented nucleus seen in CA3 region of disease (NaF only) group. In normal control and treatment groups arrows indicate neurons present are healthy with pale and round nucleus, well defined nuclear boundary and prominent nucleoli. Compared with disease group (NaF only), there is reduction of degenerated neurons in dentate gyrus areas of normal control and treatment groups

**Fig. 16:**
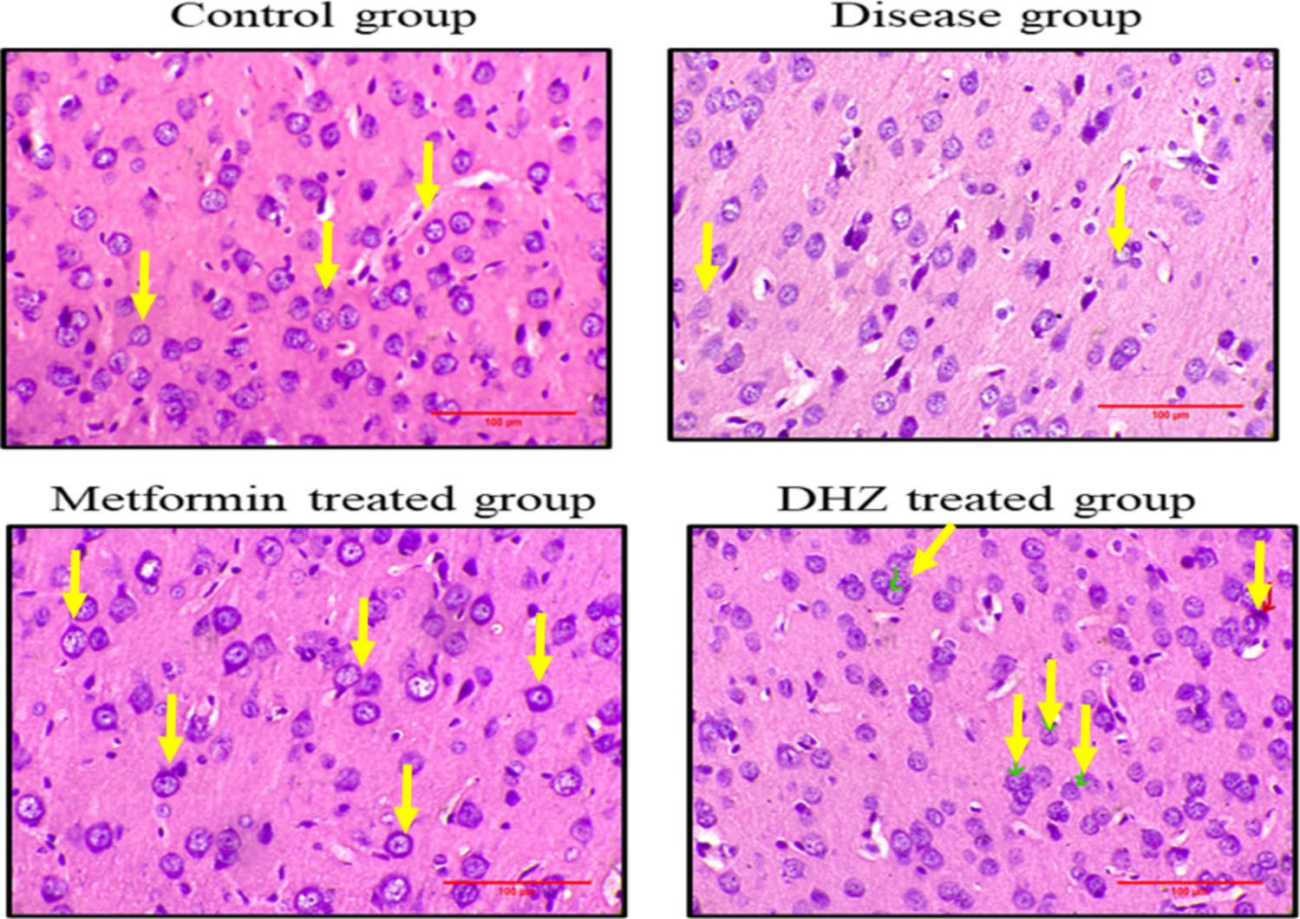
Photomicrographs of frontal cortex in different groups (H&E stain). Arrows indicating degenerated neurons in disease group (NaF only) seen in frontal cortex and no degenerated neurons (pale and round nucleus, well defined nuclear boundary and prominent nucleoli) in normal control and treatment groups

Cornu ammonis region 3 (CA3) (Fig. 13), dentate gyrus (Fig. 15) regions of hippocampus and frontal cortex (Fig. 16) showed the presence of few degenerated neurons which are darkly (basophilic) stained, with shrunken and fragmented nucleus in disease group (NaF only) (Fig. 13). Normal control and treatment groups showed the presence of neurons which were healthy with pale and round nucleus, well defined nuclear boundary and prominent nucleoli. Compared with disease group (NaF only), there is reduction of degenerated neurons in CA3, dentate gyrus areas of hippocampus and frontal cortex of normal control and treatment groups. Whereas Cornu ammonis region 1 (CA1) (Fig. 14) showed no much histological changes and there was presence of healthy neurons in all groups (Fig. 13, 14, 15, 16).

## Discussion

The findings of the present study demonstrated NaF induced oxidative damage, altering the behavior of animals, histopathology and neurochemical changes significantly. Treatment with metformin and DHZ decreased the oxidative stress and restored the antioxidant enzymes significantly. The behavioral and histopathological changes that occurred due to NaF treatment were restored in the treatment groups. This study reported the protective role of metformin and DHZ against 100 mg/L of NaF induced behavioral changes, histopathological changes and oxidative stress in brains of pre-natal rats.

Previous studies on metformin and DHZ reported multiple activities like antioxidant, anti-inflammatory and neuroprotective effects (Begum F et al. 2023). Clinical studies showed that chronic use of metformin among diabetic patients led to good cognitive function, when compared to patients who were on other anti-diabetic drugs (Kuo PC et al. 2005; Piwkowska et al. 2010; Rao MC et al. 2010; Park DB 2015; De Oliveira et al. 2016b; Dehkordi et al. 2019). Some studies have also shown that Metformin decreases AChE activity in the brain leading to restoration of normal memory and learning functions (Esteghamati et al. 2013; Saliu JA et al. 2016). Metformin and DHZ have been reported for their neuroprotective activity but there are no reports published on metformin and DHZ against exposure of NaF in prenatal pup’s developing brain. According to a study by Wang et al. in 2004, excess fluoride exposure led to diminished intelligence quotient in children (Wang J 2004). A study showed that NaF intoxication in rats led to memory loss and disability in learning (Ge Y et al. 2005). A study on pre-natal rats reported loss of memory and learning disability with increase in latency period in NaF group compared to normal control group pups (Mesram et al. 2017b).

Long term exposure to NaF decreases the spatial and episodic memory in rats (Liu et al. 2014)**.** This study assessed the episodic memory using novel object recognition test. NaF exposure in disease group significantly reduced the recognition index i.e. ability of the rats to recognize novel object in presence of a familiar object during the test phase, as compared to the normal control group (at p ≤ 0.001). Recognition index in groups treated with Metformin and DHZ was significantly increased (at p ≤ 0.0001) as compared to NaF treated group. Metformin and DHZ treated rats showed significant improvement in discrimination index wherein both treatments showed equally significant increase. These results indicate loss of recognition and episodic memory in rats exposed to NaF during their developmental stages and reversal of this impairment due to treatment with both Metformin and DHZ.

NaF decreases the motor exploratory activity which is associated with decreased levels of AChE in brain (Vani et al. 2000a; Kumar et al. 2020). Variables like decreased locomotor activity, less time spent in central square, increased latency to first line crossing, increased rearing behaviour etc. provides a measure of anxiety in the animals (Sestakova et al. 2013; Oyagbemi et al. 2020) The parameters of motor exploratory activity viz., latency, grooming, rearing, sniffing, crossing was analyzed in the open field test and the locomotor activity was analyzed using Actophotometer. The study results indicate a decrease in locomotor activity and increased anxiety in rats exposed to NaF in pre-natal model and subsequent reversal due to treatment with Metformin (p ≤ 0.05). DHZ (p ≤ 0.01) was able to partially improve the impairments in group IV.

Latency to first line crossing was significantly elevated in disease group compared to the normal control group (p ≤ 0.01). Treatment with metformin (p ≤ 0.006) was able to reduce it significantly but the difference between DHZ group and disease was not significant (p < 0.05).

NaF treatment in rats significantly increased AchE levels in hippocampus, however, increase in enzyme levels in frontal cortex was not found to be significant compared to the normal control group (p < 0.05). Metformin has been studied for its action on AchE activity in streptozotocin (STZ) induced diabetes models. Studies reported that treatment with Metformin significantly reduced AchE levels in the brain. A similar observation was made in this study where elevation of AchE levels due to NaF exposure, was significantly reversed by Metformin & DHZ treatment (p ≤ 0.001) in hippocampus of rats. However in frontal cortex, the decrease in AchE levels was not found to be significant (p < 0.05) (Saliu JA et al. 2016; Bhutada 2011) These results indicate elevated AchE enzyme levels could be involved in memory and locomotor impairments seen in various behavioural tests conducted and subsequent improvement due to treatment of Metformin and DHZ.

Lipid peroxidation (MDA levels) was significantly increased in hippocampus and treatment with Metformin was able to reduce this significantly. However, DHZ was unable to show a similar level of reduction and the difference between disease group and DHZ group was found to be non-significant at p < 0.05. In frontal cortex, MDA levels were increased in disease group compared to normal control group and DHZ (p < 0.01) decreased MDA levels significantly as compared to Metformin (p < 0.01). Results obtained for reduced GSH levels were not similar for hippocampus and frontal cortex. In hippocampus, chronic exposure to NaF, declined GSH levels compared to the normal control group (p < 0.01) and treatment with both Metformin and DHZ (p < 0.001) was not able to significantly restore its levels. In frontal cortex, however, more promising results were observed and treatment with metformin (p < 0.001) significantly increased GSH levels compared to disease group. DHZ did not show any significant changes in GSH levels.

Kumar et al. 2017 and Shanmugam et al. 2018 reported that exposure to sodium fluoride led to a reduction in body weight of rats (Shanmugam et al. 2018; Kumar et al. 2020). A similar observation was made in this study where, in the pre-natal model, a significant decrease in body weights of rats in disease group was observed compared to the normal control group (p < 0.05). It was also observed that treatment with Metformin and DHZ was able to attenuate this loss in body weight significantly (at p ≤ 0.0001).

Histopathological studies revealed that the regions of the hippocampus and frontal cortex (Fig. 16), dentate gyrus (Fig. 15), and CA3 (Fig. 13) displayed a small number of degenerated neurons that were darkly stained (basophilic), with a shrunken and fragmented nucleus in the disease group (NaF only). In contrast, the normal control and treatment groups displayed neurons that were in good health, with a pale and round nucleus, a well-defined nuclear boundary, and prominent nucleoli. The deteriorated neurons in the CA3, dentate gyrus regions of the hippocampus, and frontal cortex of the normal control and treatment groups are less than those in the disease group (NaF alone). In contrast, CA1 (Fig. 14) in all groups had healthy neurons and did not exhibit many histological alterations.

An additional study was conducted for the rats in prenatal model in which the pups were tested for normal reflexes in early post-natal days (day 7–11) to check whether administration of NaF during gestation period had any effect on the reflexes and if treatment with metformin and DHZ were able to show any improvement in these reflexes. In the present study, no significant effect of NaF in prenatal rats was observed on any reflex. Also, treatment with Metformin and DHZ did not significantly improve the performance of pups for any reflex compared to both normal control and disease group. Thus, it can be concluded that NaF administration during gestation period does not have any effect on formation of reflexes in rat pups (Rao MC et al. 2011; Park DB 2015).

## Conclusion

NaF exposure did not significantly impact neonatal reflexes, but it did impair cognitive function. Also led to decreased locomotor activity and increased anxiety-like behavior in pups. Metformin and DHZ treatments significantly improved cognitive deficits and reduced anxiety, with Metformin being particularly effective in restoring normal locomotor activity levels. Both Metformin and DHZ showed potential in mitigating the biochemical alterations, pressing on their antioxidative properties. Neuronal degeneration in the hippocampus of NaF-exposed rats, was effectively prevented by Metformin and DHZ treatment.

## Data Availability

Data available upon request.
